# State-of-the-Art Review of Studies on the Flexural Behavior and Design of FRP-Reinforced Concrete Beams

**DOI:** 10.3390/ma18143295

**Published:** 2025-07-12

**Authors:** Hau Tran, Trung Nguyen-Thoi, Huu-Ba Dinh

**Affiliations:** 1 Laboratory for Computational Civil Engineering, Institute for Computational Science and Artificial Intelligence, Van Lang University, Ho Chi Minh City 70000, Vietnam; ba.dinhhuu@vlu.edu.vn; 2 Faculty of Civil Engineering, School of Technology, Van Lang University, Ho Chi Minh City 70000, Vietnam; 3 Laboratory for Applied and Industrial Mathematics, Institute for Computational Science and Artificial Intelligence, Van Lang University, Ho Chi Minh City 70000, Vietnam; trung.nguyenthoi@vlu.edu.vn; 4 Faculty of Mechanical-Electrical and Computer Engineering, School of Technology, Van Lang University, Ho Chi Minh City 70000, Vietnam

**Keywords:** artificial intelligence/machine learning, discrete element analysis, design standards, finite element analysis, FRP RC beams

## Abstract

Fiber-reinforced polymer (FRP) bars have great potential to replace steel bars in the design of reinforced concrete (RC) beams since they have numerous advantages such as high tensile strength and good corrosion resistance. Therefore, many studies including experiments and numerical simulations have focused on the behavior of FRP RC beams. In this paper, a comprehensive overview of previous studies is conducted to provide a thorough understanding about the behavior, the design, and the limitations of FRP RC beams. Particularly, experimental studies on FRP RC beams are collected and reviewed. In addition, the numerical analysis of FRP beams including the finite element (FE) analysis, the discrete element (DE) analysis, and artificial intelligence/machine learning (AI/ML) is summarized. Moreover, the international standards for the design of FRP RC beams are presented and evaluated. Through the review of previous studies, 93 tested specimens are collected. They can be a great source of reference for other studies. In addition, it has been found that the studies on the continuous beams and deep beams reinforced with FRP bars are still limited. In addition, more studies using DE analysis and AI/ML to analyze the response of FRP RC beams under loading conditions should be conducted.

## 1. Introduction

Traditionally, concrete is an important material that is utilized broadly in the construction industry and is studied extensively in literature [1,2,3,4,5,6,7,8,9,10,11,12]. To improve the tensile behavior of concrete structures, the application of concrete normally goes with the implementation of steel reinforcement. However, the durability of steel RC structures is significantly influenced by the working environment. Particularly, when working in a harsh environment, steel reinforcement can be corroded, which induces cracks in concrete and causes a reduction in the load-carrying capacity of the steel RC structures. Therefore, the cost for the maintenance of deteriorated steel RC structures is enormous (over US$1.8 trillion annually [13]). To increase the durability of the RC structures, fiber-reinforced polymer (FRP) bars have been applied as a substitution for steel reinforcement because they have numerous superior characteristics such as high strength, nonmagnetic property, and the ability to resist corrosion. FRP bars are produced from unidirectional fibers embedded with polymer matrix. The production of FRP bars requires less energy and causes less pollution in comparison to the production of steel [13]. Therefore, it is more sustainable and environmentally friendly.

**Figure 1:**
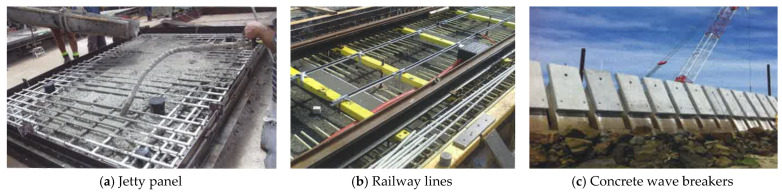
Applications of FRP in practical structures [14].

**Figure 2:**
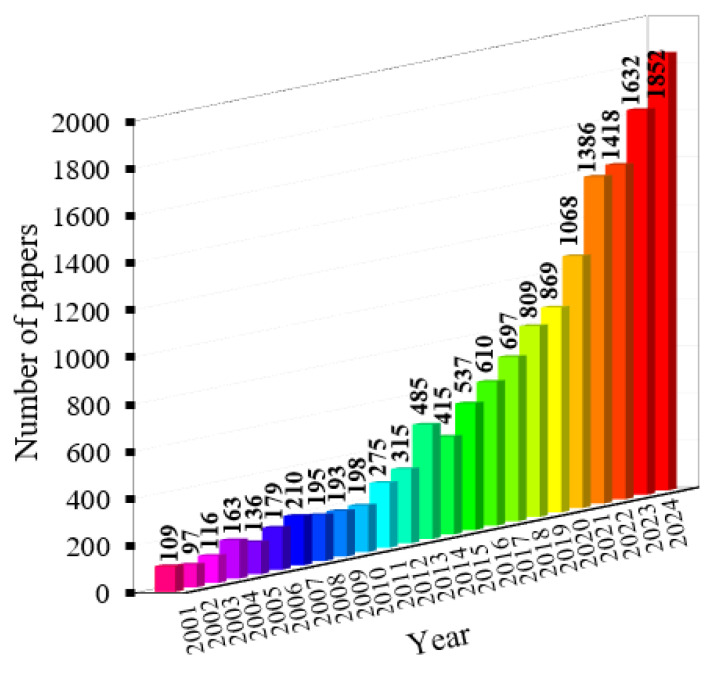
Number of studies on FRP-reinforced concrete structures [29].

FRP bars have been increasingly applied in the construction industry as a replacement of steel to cast RC components such as columns, walls, floors, and beams. Some practical structures that used FRP to reinforce concrete are presented in Figure 1 [14]. Particularly, FRP bars can be utilized to produce precast concrete panels such as jetty panel (Figure 1a) or concrete wave breakers. In addition, FRP bars can be applied to cast the concrete of railway lines as shown in Figure 1b. FRP can also be used in prestressed structural members [15]. The application of FRP can reduce the weight of concrete structures as the density of FRP is quite low (see Table 1). This is a huge advantage when constructing mega projects because the dead load can be reduced significantly. In addition, the ability to resist chemical attacks allows FRP-reinforced structures to be more sustainable. However, the price of FRP bars is quite expensive compared to that of steel bars (see Table 1), which is one of the biggest limitations of FRP reinforcement. Due to a variety of advantages and numerous practical applications, lots of studies have been conducted to investigate the behavior and the durability of FRP RC structures [16,17,18,19,20,21,22,23,24,25,26,27,28]. The number of studies on FRP RC structures has also soared to approximately more than 16 times from 2001 to 2024 as shown in Figure 2 [29]. Among those studies, the research on FRP RC beams has drawn great attention from researchers [30,31,32,33,34,35,36,37,38,39,40,41,42,43,44,45,46,47,48,49,50,51]. These are numerical and experimental studies that provide a broad range of knowledge in FRP RC beams.

Therefore, a comprehensive study should be conducted to summarize the current literature about FRP RC beams, which can become a useful source of knowledge for future studies in this field. As a result, this research aims at conducting a comprehensive overview about studies on the flexural behavior and design of FRP RC beams. Based on the methodology, information about the investigated specimens, used materials, type of applied software, and the findings of the research, studies are collected and classified. Firstly, the experimental studies conducted to analyze the response of FRP RC beams under loading conditions are collected and reviewed. Secondly, numerical studies including the finite element (FE) analysis, the discrete element (DE) analysis, and artificial intelligence/machine learning (AI/ML) are summarized. Thirdly, the design standards of FRP beams are presented and the comparison between standards is also carried out. Finally, the research gaps are deducted, and future research directions are recommended.

## 2. FRP-Reinforced Concrete (RC) Beams

**Figure 3:**
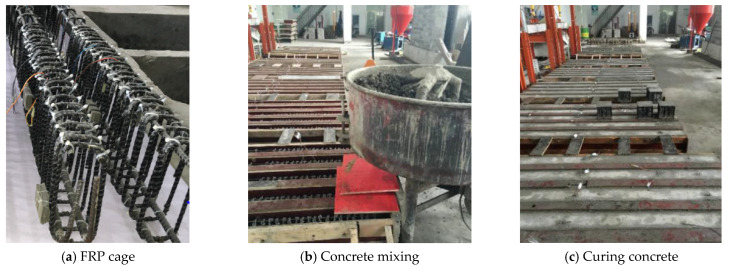
Casting FRP-reinforced concrete beams [52].

FRP RC beams are cast by pouring concrete into a cage assembled from fiber-reinforced polymer (FRP) bars as depicted in Figure 3 [52]. Based on the structural requirements, the environmental requirements, the availability of the materials in the market, and the budget, a suitable type of FRP and concrete will be selected for the design. In this part, the characteristics of each type of FRP, the features of some common kinds of concrete, the bond between FRP/concrete interface, and the failure mechanism of FRP-reinforced concrete beams are presented.

### 2.1. Fiber-Reinforced Polymer (FRP)

**Figure 4:**
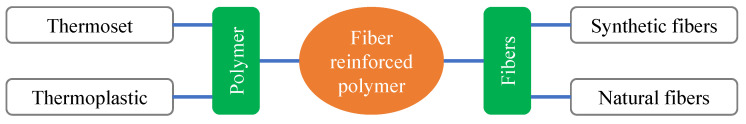
Components of fiber-reinforced polymer (FRP).

FRP is a kind of composite material that comprises two main components including continuous fibers and a polymer matrix as depicted in Figure 4 [53]. The continuous fibers are attached together by the polymer matrix to manufacture FRP products in the form of bars, sheets, laminates, or tubes. These products are considered brittle materials that have linear stress—strain relationship. There are two major types of polymer matrices: thermoset and thermoplastic. However, the application of thermoplastic matrices in practice is quite limited as they do not have good mechanical and physical properties. Based on the types of fibers, FRP can be classified into four prevalent categories. These types include glass fiber-reinforced polymer (GFRP), carbon fiber-reinforced polymer (CFRP), basalt fiber-reinforced polymer (BFRP), and aramid fiber-reinforced polymer (AFRP) as shown in Table 1. As can be seen from this table, CFRP has higher young modulus than other types of FRP, but it is the most expensive material. GFRP has lower young modulus than CFRP, but the tensile strength is high and the price is reasonable compared to BFRP and CFRP. Therefore, it is applied widely in practice and the number of specimens reinforced with GFRP bars accounts for 72%. This table also illustrates that steel bars can be cheaper than FRP bars, but they have lower tensile strength and higher density.

There are various types of glass fibers [54,55]. Each type of glass fiber has its own typical physical and mechanical properties. The tensile strength and the elastic modulus of glass fibers can be up to 4.9 GPa and 86.9 GPa, respectively [55,56]. These numbers demonstrate that glass fibers have much higher tensile strength than conventional steel reinforcing bars, but they have much lower density and elastic modulus. In the case of A-glass fiber, it has higher durability, strength, and electrical resistivity. Type C-glass fiber is better at resisting corrosion. Type D-glass fiber has low dielectric constant. Type E-glass fiber has higher strength and electrical resistivity. Type AR-glass fiber is capable of Alkali resistance. Type R-glass fiber has higher strength and acid corrosion resistance. Regarding type EGR-glass fiber, it has high tensile strength and large elastic modulus. Finally, type S2-glass fiber has high strength, modulus, and stability. More details about the physical properties of glass fibers are illustrated in Table 2. Other studies about the properties of GFRP have been carried out [57,58,59,60,61,62,63,64].

CFRP is widely applied in numerous fields including the construction industry, the fabrication of robot parts, the aerospace industry, and the production of cars due to its noticeable merits such as its high strength, high elastic modulus, alkaline resistance, and light weight [54,65,66,67]. A CFRP composite can have a tensile strength and an elastic modulus of up to 3.9 GPa and 784 GPa, respectively [65]. With these superior mechanical properties along with the high strength-to-weight ratio, CFRP has great potential to replace steel in the construction field. However, the cost of CFRP is relatively expensive due to the manufacturing procedure [54,65]. If this disadvantage can be eliminated, the application of this kind of material in practice will dramatically rise. Because of the great potential of CFRP, many studies have been carried out to explore its mechanical properties [64,67,68,69,70,71,72,73].

The main component of BFRP is basalt fiber which is a kind of natural fiber made from basalt rocks. Basalt fibers can have tensile strength and elastic modulus of up to approximately 4.84 GPa and 110 GPa, respectively [27]. Due to its numerous advantages and reasonable price, BFRP has many applications and can be a good selection besides CFRP and GFRP [27,54]. As a result, many studies have been conducted to investigate the mechanical properties of BFRP under different environmental factors [27,74,75,76,77,78,79,80,81,82,83]. The studies by [68,84] explored that the tensile strength of BFRP could decrease in a chemical environment. In addition, they also found that basalt fibers had excellent performance at elevated temperatures since it retained almost 90% of the initial tensile strength after being exposed to high temperature. Wang et al. [82] investigated the chemical durability and mechanical properties of BFRP by boiling it in the distilled water, sodium hydroxide, and hydrochloric acid. The results of their study showed that the flexural strength and the modulus of BFRP could be reduced in a chemical environment. Lopresto et al. [79] conducted mechanical tests to assess whether BFRP could substitute GFRP in practical applications. They discovered that BFRP possessed excellent mechanical characteristics in terms of elastic modulus, strength, and impact force, which illustrated that BFRP could be used to replace GFRP.

AFRP is fabricated from aramid fibers that are synthetic fibers made from the polyamide family. The tensile strength and the elastic modulus of AFRP can be up to 3.6 GPa and 175 GPa, respectively [65]. Although the tensile strength and the modulus are relatively high, AFRP has several disadvantages such as low compressive strength and it is sensitive to high temperature and moisture, which limits its practical application [85,86].

### 2.2. Major Types of Concrete

There are several popular kinds of concrete that can be used for casting FRP RC beams. Generally, ordinary Portland cement concrete (OPC)/normal concrete, which is the mixture of water, river sand, cement, and gravel, is the most common kind of concrete used in the construction industry since its components are abundant in the market. In addition, the strength of normal concrete can be controlled by changing the ratio of ingredients or by using additives. In recent decades, various new types of concrete have been utilized. Based on the ingredients and types of aggregates, four prevalent types of concrete including lightweight aggregate concrete (LWAC), ultra-high performance concrete (UHPC), sea-water sea sand concrete (SWSSC), and geopolymer concrete (GPC) are introduced in this section. These concrete types have been used in many studies about FRP-reinforced concrete beams (see Table 3). More details about the characteristics of each type of concrete are presented below.

**Figure 5:**
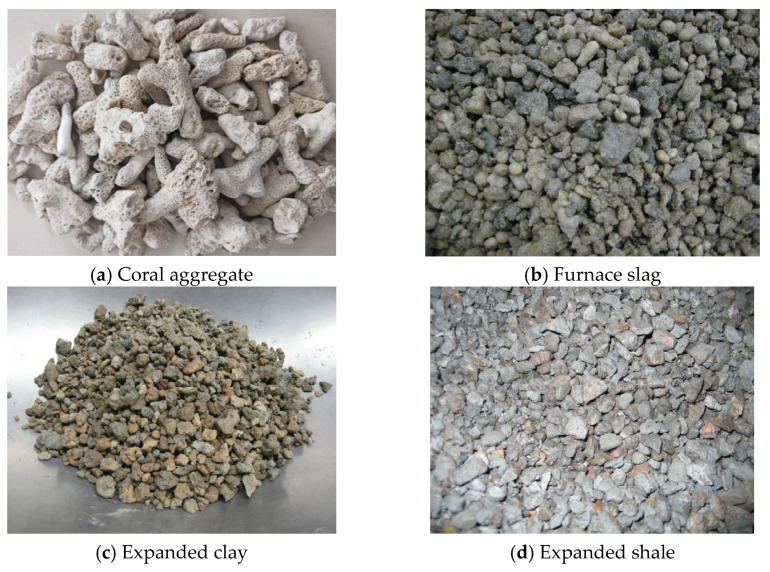
Lightweight aggregates [87,89].

Many studies related to LWAC have been found [34,50,87,88,89,90,91]. The main ingredients of this type of concrete are lightweight aggregates such as furnace slag, expanded clay, expanded shale, and coral aggregate (Figure 5) mixed with water, cement, fly ash, and silica fume. LWAC has many advantages including thermal insulation, fire resistance, and being light weight, which can contribute significantly to the reduction in dimensions of structures. However, there are also drawbacks related to mechanical properties that can strongly influence the performance of LWAC structures such as high creep, high brittleness, and high shrinkage [87,89]. Therefore, more studies on the behavior of structural elements cast by LWAC should be conducted.

**Table 3:** Major types of concrete used in previous studies of FRP RC beams.

Source	Type of Concrete	Key Findings
[16,26,92,93,94,95]	OPC	ACI 318 underestimated the deflection of FRP RC beams up to 70%. The proposed model could predict the deflection of FRP beams with an error of less than 10%. ACI 440.1R overestimated crack widths but underestimated the deflection of FRP beams. FRP RC beams experienced large deformation to achieve predicted moment capacity.
[96,97,98]	UHPC	The load and deflection at the first crack were not dependent on the reinforcement ratio. ACI 440.1R overestimated the deflection of FRP-reinforced UHPC beams. UHPC could improve the ductility of the beam because of its high compressive strain and high tensile strength. The ductility model of FRP normal concrete beams could not be used for FRP UHPC beams.
[45,48,49,52]	SWSSC	GB 50608 underestimated the moment capacity of FRP beams. The moment capacity of FRP-reinforced SWSSC beams increased when shear/span ratio was reduced. Compression-cast FRP beams have higher cracking instances due to the higher strength of concrete. With the same applied loads, the maximum crack with compression-cast FRP beams is smaller than that of FRP OPC beams. A model for predicting the ultimate moment was proposed. A cracking moment is governed by the tensile strength of concrete. Both ACI 440.1R and GB 50608 should be modified to predict the deflection of FRP beams better.
[33,38,40,51,99,100]	GPC	Both ACI 440.1R and CSA S806 underestimated the ultimate moment of FRP GPC beams. It was better to use CFRP than GFRP, BFRP, and AFRP as tensile reinforcement of concrete beams. GPC could be an alternative material to replace traditional concrete. Beams with steel stirrups had better performance than beams with FRP stirrups. GPC had higher compressive strain than normal concrete. ACI 440.2R underestimated the experimental load of the GFRP-reinforced T-beam. The structural behavior of geopolymer concrete was similar to normal concrete.
[34,50,88]	LWAC	CFRP bars could be a good option to replace steel reinforcement. The proposed equations to predict the ultimate moment of FRP-reinforced LWAC could give reliable results compared to experiments. Research on anchoring measures at ends of CFRP bars should be conducted. A model to predict the crack width of CFRP LWAC beams was proposed. GFRP LWAC beams had large deflection and crack width. The flexural capacity of BFRP LWAC beams could be predicted by specifications for FRP-reinforced OPC beams. The ultimate moments of BFRP LWAC beams reduced significantly when exposed to high temperature.

UHPC is typically a combination of cement and silica fume that is mixed with relatively low water-to-cement ratio (normally less than 0.25), superplasticizers, and ultra-fine silica sand [101,102]. Unlike conventional concrete, UHPC can be considered as a type of mortar since coarse aggregates are not used in the mixture. In addition, this type of concrete has numerous superior advantages in comparison with conventional concrete. Particularly, UHPC has very high compressive strength with a minimum value of 150 MPa, excellent long-term durability, and strong ability to resist environmental factors [96,102]. As a result, this type of concrete is applied broadly in the construction industry from bridges to structural components of residential buildings. The application of UHPC to cast FRP RC beams has also been found in some studies [96,97,98,103]. Despite having many merits, a critical disadvantage of UHPC is that it is a brittle material that can fail suddenly under loading conditions. Hence, many studies have been carried out to increase the ductility of this type of concrete by adding steel/composite fibers or by optimizing the mix using limestone powder, metakaolin, nano-silica, zeolite, and lithium slag [102].

In SWSSC, sea sand and sea water are used to replace river sand and normal water, respectively. This is a good approach to deal with the shortage of river sand occurring in many countries. This type of concrete is not very different from conventional concrete, but the chloride ions in the sea water can contribute significantly to the corrosion of steel reinforcement in concrete structures [52,104]. As a result, FRP bars are applied to replace traditional steel reinforcement, and many studies about FRP-reinforced SWSSC beams have been conducted [45,48,49,105].

Geopolymer concrete is a type of green concrete that utilizes alkali-activated cementitious materials to replace traditional Portland cement. In addition, other ingredients used to mix geopolymer concrete are aluminosilicate materials also known as solid wastes collected from industrial production processes such as metakaolin, slag, fly ash, and blast furnace [50,51]. In addition, this kind of concrete also has various advantages such as chemical resistance, low carbon emission, and temperature resistance. Therefore, geopolymer concrete is very friendly to the environment and it is an ideal material for the development of sustainable construction. As a result, geopolymer concrete is employed widely to cast FRP RC beams, and has been investigated in many studies [38,40,100].

### 2.3. Concrete Stress–Strain Relationship

The compressive stress–strain relationship of concrete has been investigated and proposed in some studies [28,106,107,108,109,110]. This relationship is extremely important when conducting numerical simulations of concrete structures. In Table 4, several typical concrete compressive stress–strain models obtained from previous studies are summarized.

### 2.4. Bond Between FRP Bars and Concrete

The bond at the interface between concrete and FRP bars plays a crucial role in the behavior of FRP RC beams as it can significantly affect the load-transferring mechanism between concrete and FRP. This bond behavior is represented by a bond stress–slip relationship that has been investigated in some studies [111,112,113,114,115]. Some typical bond stress–slip relationships were proposed in the studies by [112,115] and [114] as shown in Equations (1) and (2), respectively.

(1) $$\tau =\tau_{\max}{\left[1-e^{4s}\right]}^{0.5}$$

(2) $$\tau =\left\{\begin{array}{l} \tau_{\max}{\left(\frac{s}{s_{m}}\right)}^{\alpha }\;\;\;\;\;\;\mathrm{if}\;0\leq s\leq s_{m}\\ \tau_{\max}-\left(\tau_{\max}-\tau_{2}\right)\frac{s-s_{m}}{s_{2}-s_{m}}\;\mathrm{if}\;s_{m}<s\leq s_{2}\\ \tau_{2}\;\;\;\;\;\;\;\;\;\;\;\mathrm{if}\;s_{2}<s \end{array}\right.$$

Several factors can influence the bond strength between concrete and FRP including the FRP bar diameter (*d_bar_*), the thickness of concrete cover (*C*), the compressive strength of concrete (*f_c_*), bond length (*l_e_*), and the surface of FRP bars. Among these parameters, the surface of FRP bars and the value of fc are the two most influential factors. Nepomuceno et al. [111] proposed an equation as shown in Equation (3) to determine the bond strength on the basis of fc and FRP bar surface conditions represented by α and *β*. The values of α and *β* for different FRP surfaces are given in Table 5. In some studies [113,116], the effects of other factors are also accounted as given in Equations (4)–(6). The slip (*s_m_*) corresponding to the bond strength can be calculated by Equations (7) and (8).

(3) $$\tau_{\max}=\alpha {\left(f_{c}\right)}^{\beta }$$

(4) $$\tau_{\max}=\frac{13.5\times {\left(f_{c}\right)}^{0.5}}{d_{bar}}$$

(5) $$\tau_{\max}=\frac{14.7\times {\left(f_{c}\right)}^{0.5}}{d_{bar}}$$

(6) $$\tau_{\max}=0.083\times {\left(f_{c}\right)}^{0.85}\times \left(4.0+0.3\times \frac{C}{d_{bar}}+100\times \frac{d_{bar}}{l_{e}}\right)$$

(7) $$s_{m}=0.011d_{bar}\times \left(\frac{2}{\frac{f_{c}}{33}+1}\right)\times \frac{d_{bar}}{h}\times {\left(\frac{l_{e}}{d_{bar}}\right)}^{1.6}$$

(8) $$s_{m}=\left(\frac{\tau_{\max}}{f_{c}}\right)\times \left(\frac{l_{e}}{60}\right)$$

### 2.5. Failure Mechanism of FRP RC Beams

The flexural failure mechanism of FRP RC beams is strongly dependent on the constitutive laws of concrete in compression and the constitutive laws FRP bars in tension. Since the stress–strain relationship of FRP bars is linear and the ductility in the behavior of concrete is relatively low, the failure of FRP beams is classified as brittle failure, which includes two major failure modes: FRP rupture and concrete crushing. It should be noted that concrete crushing is generally the preferred failure mode in the design of FRP RC beams as the failure by FRP rupture is very brittle and it can fail suddenly without any signs. In contrast, the failure of concrete beams reinforced with steel bars is much more ductile. The reason for this difference can be attributed to the elastic–plastic behavior of steel bars. Based on this property of steel reinforcement, the preferred failure mode of steel RC beams is steel yielding followed by concrete crushing due to the propagation of cracks towards the concrete compression zone.

**Figure 6:**
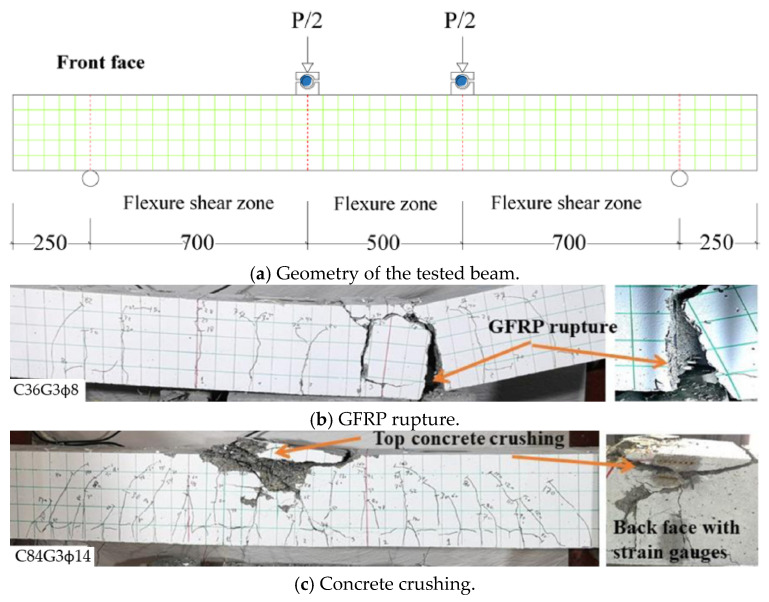
Failure modes of FRP RC beams tested by [26].

**Figure 7:**
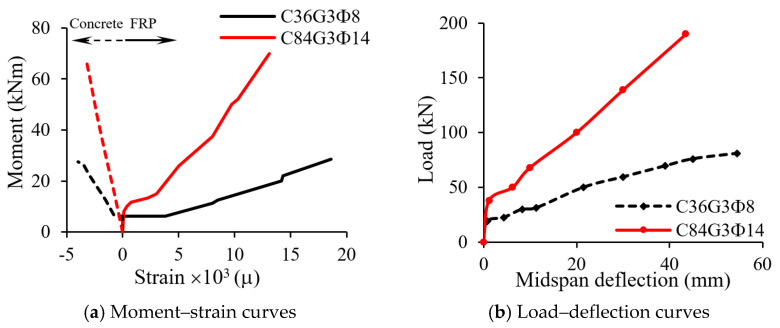
Test results obtained from [26].

Figure 6 demonstrates a typical four-point bending test conducted to investigate the failure of FRP RC beams [26]. Two specimens in their experiment, namely C36G3Φ8 and C84G3Φ14, are presented. The type of FRP bars used in these specimens is glass fiber-reinforced polymer (GFRP) with the ultimate strain of 1.66%. The failure mode of beam C36G3Φ8 and beam C84G3Φ14 is GFRP rupture and concrete crushing, respectively, as can be observed from Figure 6b,c. These failure modes can also be identified by comparing the GFRP strain obtained from the experiment (Figure 7a) with the ultimate GFRP strain. In Figure 7b, the load–deflection relationships of beam C36G3Φ8 and beam C84G3Φ14 indicates that the failure of FRP RC beams is brittle as the beams fail suddenly after reaching the peak load.

## 3. Experimental Studies on FRP RC Beams

**Figure 8:**
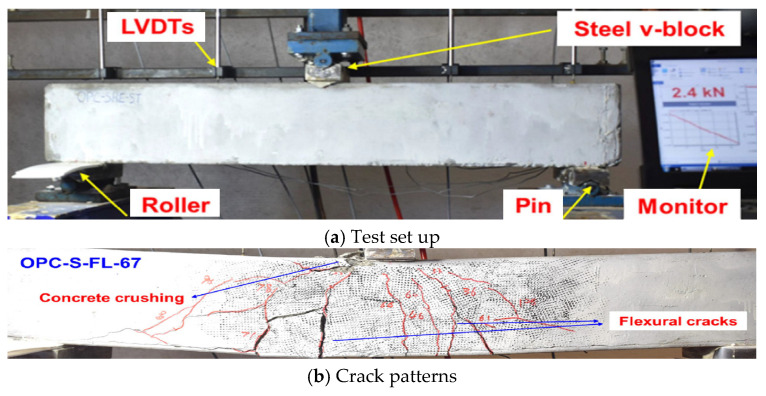
Three-point bending test of FRP RC beams [41].

FRP bars have been applied broadly to reinforce concrete beams due to their various advantages as mentioned above. Hence, many experimental studies have been conducted using the three-point bending test (Figure 8) and the four-point bending test (Figure 6) to investigate the flexural behavior of FRP RC beams. However, the four-point bending test is prioritized as it can demonstrate more clearly the details of the failure mechanism such as concrete crushing, FRP rupture, cracking patterns in the pure flexural zone, and cracking patterns in the flexural–shear zone. These studies aim at various objectives by considering different key parameters (Table 6). In this section, these experimental studies are collected and summarized.

### 3.1. Beams Reinforced with GFRP Bars

Alsayed [16] tested four series of simply supported beams named as A, B, C, and D to explore the behavior of GFRP RC beams and to compare the performance of these beams with steel RC beams. Series A included control specimens that were reinforced with steel bars. Series B, C, and D comprise concrete beams reinforced with GFRP bars. According to the results of his study, the mid-span deflection of GFRP beams was two times higher than that of the steel-reinforced beams, which was 10.64 mm compared to 5.44 mm. In addition, at the same service load and deflection as those of the control beams in series A, the ultimate loads of beams in series C and D were 36% and 53% higher than those of series A. This study also pointed out that the formulas of ACI 440.1R standard underestimated the deflection of GFRP RC beams.

Theriault and Benmokrane [47] carried out experiments on GFRP-reinforced concrete beams to discover the influence of FRP reinforcement ratio and the compressive strength of concrete on the crack formation of the tested beams. The results of their study demonstrated that the compressive strength of concrete had negligible effects on the width and the spacing of cracks. However, the reinforcement ratio could significantly reduce the crack width and height. Their study also mentioned that the energy dissipation through elastic deformation could occur when only 20–30% of the FRP bar strength is considered in the design.

Toutanji and Deng [95] tested and analyzed the deformations and cracks of three series of GFRP RC beams to evaluate the calculation equations given by ACI 440.1R design standard. Each series had two identical beams. According to the results of their study, ACI was able to predict the crack width of the tested beams reasonably. However, when the beams were reinforced with two layers of FRP bars, the formulas given in ACI were suggested to be modified to predict the crack width of FRP beams more accurately. As a result, they recommended increasing the parameter accounting for the bond between FRP bars and concrete from 1.2 to 1.4 for the ACI to predict the crack width more precisely.

Benmokrane et al. [18] investigated the flexural behavior of concrete beams reinforced with GFRP under the four-point bending test. In their studies, many parameters were considered including the type of GFRP bars, the span-to-depth ratio of the beams, and the compressive strength of concrete. Particularly, two types of GFRP were used, one of them had their surface covered by sand particles, and the other type had the deformation on the surface. In addition, three values of span-to-depth ratios including 10, 6.67, and 5.45 were obtained. The results of their study showed that it was possible to use GFRP RC beams, especially in aggressive environments, to replace steel bars. The optimal design of GFRP beam could be achieved when a reasonable reinforcement ratio and sectional height-to-depth ratio were used. The study also recommended that the formulas given in ACI 440.1R should be reassessed since it overestimated the moment capacities of the tested beams. Hence, the method for predicting the flexural capacity of FRP RC beams were studied in several research [119].

Lau and Pam [120] conducted the experiment on pure GFRP RC beams and hybrid steel–GFRP RC beams to investigate their flexural performance. According to their study, both hybrid beams and pure over-reinforced GFRP beams demonstrated higher ductility than that of under-reinforced GFRP beams. To take advantage of steel bars and to avoid brittle failure, their study recommended that the hybrid beam should be designed properly to make sure that steel yielding would occur first, following by the concrete crushing and FRP rupture. Also based on the results of their test, they suggested that the minimum FRP reinforcement required by ACI 440.1R could be reduced by approximately 25%.

Maranan et al. [99,121] conducted experiments to discover the behavior of geo-polymer concrete beams reinforced with GFRP bars and GFRP-steel bars. The effect of various parameters such as the diameter of FRP bars, the reinforcement ratio and the anchorage system on the flexural capacity and the serviceability performance of the beams under four-point bending was also investigated. Their study found that in the case of concrete crushing failure mode, the ultimate moment of GFRP-reinforced geo-polymer concrete beams was 1.2 to 1.5 times higher than that of steel-reinforced geo-polymer concrete beams if they have the same reinforcement ratio. In addition, the increase in the reinforcement ratio led to better serviceability performance of the beam, higher stiffness and smaller crack width. The actual concrete crushing strain obtained from their experiments was from 0.0042 to 0.0048, which was higher than those recommended in ACI 440.1R and CSA S-806 (0.003 and 0.0035, respectively).

Escorcio and Franca [118] carried out experiments to assess the possibility of using GFRP bars to replace the corroded steel reinforcements in concrete beams. The tested beams were cast in two phases to reflect the rehabilitation process of corroded steel-reinforced concrete beams. The tested beams were divided into two groups: group REHABGFRP1 had beams with similar reinforcement ratio and ultimate moment to referenced beams, and group REHABGFRP2 had beams with similar displacement to referenced beams. It was observed from their study that the deflection of beams in group REHABGFRP1 was 1.8 times higher than that of the referenced beam even though they had similar loading capacity. In addition, with the same deflection, the loading capacity of beams in group REHABGFRP2 increased by approximately 215.5% compared to that of the referenced beam. The results of their study also showed that GFRP could be used to rehabilitate corroded steel RC beams since the beams rehabilitated with GFRP were able to achieve the original loading capacity or deflection.

El-Nemr et al. [36] investigated the flexural strength and serviceability of GFRP beams by testing full-scale beams with the dimension of 4250 mm long, 200 mm wide, and 400 mm thick. The results of the study showed that higher reinforcement ratio led to higher load-carrying capacity, lower deflection, and smaller crack width. Their study also illustrated that sand-coated GFRP bars exhibited good bonds with the surrounding concrete due to its uniform surface and the absence of discontinuity points. In addition, the relationship between strain and crack width was examined. When the strain of GFRP bars was 2 × 10^3^ micro-strain, the width of cracks of GFRP beams was less than 0.5 mm, which was the same as the suggestion of ISIS.

### 3.2. Beams Reinforced with CFRP Bars

Thiagarajan [94] conducted bond test and strength test to investigate the flexural behavior of sandblasted CFRP RC beams under four-point bending. The results of his study showed that the bond between sandblasted CFRP bars and concrete was not an issue since the failure did not appear during the pullout test. In addition, the CFRP rods demonstrated high strain to achieve high stress due to their low elastic modulus. Hence, large deformation of tested beams was observed, which could be a drawback when using FRP as reinforcement. In addition, the results of the test also showed that ACI 440.1R overestimated the crack width of the tested CFRP beams.

Kassem et al. [93] experimentally studied the influence of parameters such as the reinforcement ratio, the surface of FRP bars, and the type of FRP bars on the behavior of FRP RC beams. The results of their study showed that FRP beams behaved linearly before and after cracking. The stiffness of the beam after cracking decreased significantly. In addition, They found that the failure by concrete crushing occurred when the reinforcement ratio is larger than 1.4 times the balance reinforcement ratio, which agreed well with the classification of ACI 440.1R. Based on the observation from the experiment, sand-coated FRP bars have better bond with concrete since beams reinforced with these FRP bars have more cracks with smaller crack spacing in comparison with the beams with ribbed-surface bars. The bond factors were found in the range from 0.86 to 1.32 for the FRP bars.

Ahmed et al. [33] conducted experiments to explore the behavior of geo-polymer concrete beams and conventional concrete beams reinforced with CFRP. According to the results of their study, only 66.7% of the type of failure mode predicted by ACI 440.1R was the same as that obtained from the experiment. Hence, the guidelines given by ACI 440.1R to predict the failure mode could be reviewed. In addition, the first cracking load was improved significantly up to 40.6% when the compressive strength of concrete was increased. Their study also showed that the geo-polymer concrete beams had smaller crack width in comparison with conventional Portland concrete beams.

### 3.3. Beams Reinforced with BFRP/AFRP Bars

Although several experimental studies on RC beams reinforced with BFRP/AFRP bars have been found [25,30,35,93,122], the number of these studies is still limited when compared to those of beams reinforced with GFRP/CFRP bars. According to the study by Pawlowski and Szumigala [93,122], the design of BFRP/AFRP beams can be similar to that of GFRP beams. Based on the test of simply supported BFRP beams under four-point bending, they found that BFRP/AFRP beams behaved almost linearly until failure with large deformation. In addition, their failure modes and behavior were significantly dependent on the reinforcement ratio.

## 4. Numerical Studies on FRP RC Beams

**Figure 9:**
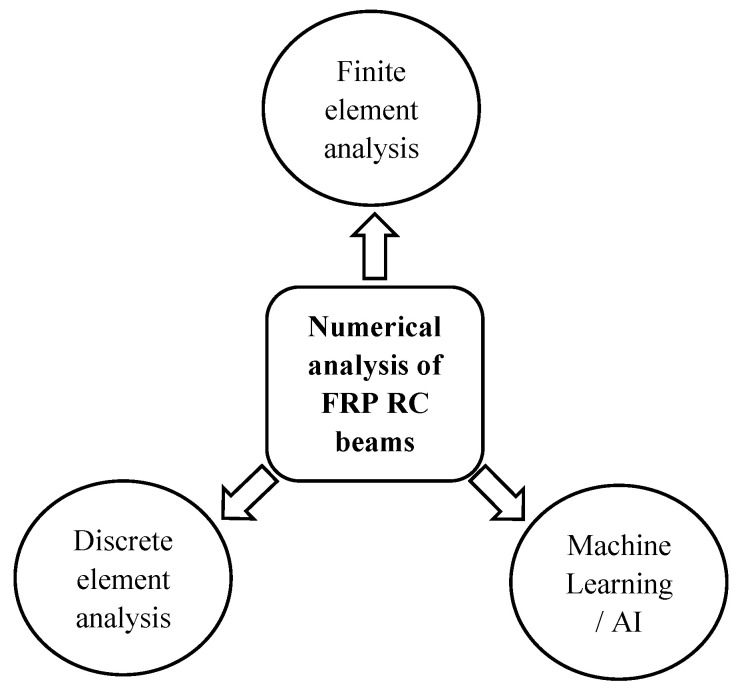
Approaches for the numerical analysis of FRP RC beams.

Conducting experiments is a practical approach to explore the behavior of FRP RC beams. However, this method is costly, and it also consumes a lot of time. Therefore, numerical models of FRP RC beams are constructed to minimize the disadvantages of the experiments and to optimize the design of practical structures. There are currently three major numerical approaches applied widely with high accuracy to analyze the response of FRP beams, which are the FE analysis, the DE analysis, and AI/ML (see Figure 9 and Table 7). By applying FE analysis and DE analysis, the behavior of FRP RC beams can be captured in detail including the deformation, the crack formation and propagation, the bond between FRP and concrete, the stress distribution, and the failure mechanism. In addition, FE analysis is the most popular method used to simulate the behavior of FRP. However, this approach usually struggles with convergence problems when modeling members with nonlinear behavior such as concrete beams. In DE analysis, convergence problems can be surpassed, but the computational time is quite a considerable issue as members should be meshed appropriately. AI/ML approach can save computational time significantly after the model is trained, and the convergence problem also does not exist in this method. Nevertheless, the accuracy of the AI/ML approach is strongly dependent on the collected database. Therefore, these three approaches are considered in this section, and studies using these numerical analysis approaches to analyze FRP RC beams are collected and summarized.

### 4.1. Finite Element (FE) Analysis

**Figure 10:**
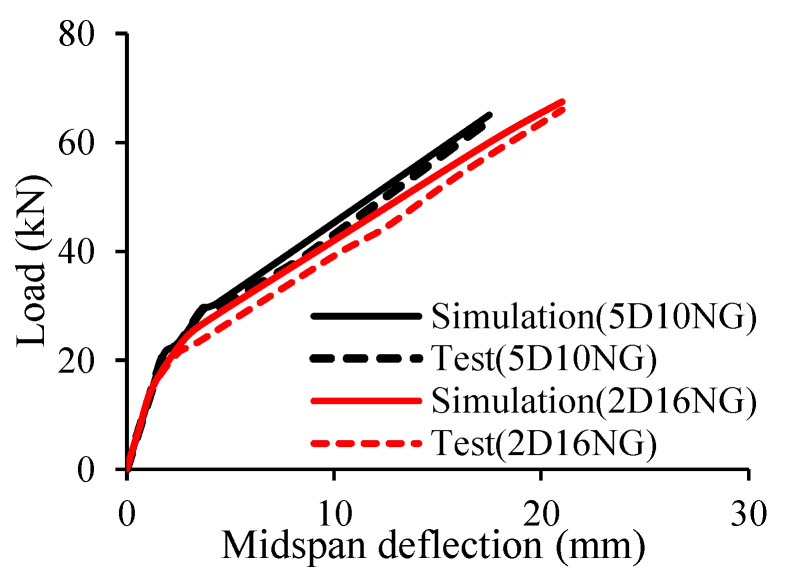
Comparison between test and simulation [42].

Kazemi et al. [42] employed 3D numerical simulation using ABAQUS to simulate the response of concrete beams reinforced with GFRP bars. Both normal and high strength concrete were investigated in their study along with the consideration of some parameters such as the flexural reinforcement ratio, and the amount of transverse reinforcement. Based on the comparison between the test and the simulation as shown in Figure 10, it can be seen that their proposed model can predict the behavior of the tested beams quite well with the mean and *CoV* of only around 0.96 and 2.69%, respectively. This figure also clearly demonstrates that the failure of FRP RC beams is quite brittle. According to their findings, transverse reinforcing bars could transfer the stress of concrete to flexural reinforcing bars, which contributed to the reduction in the mid-span deflection and crack width. In addition, the use of high strength concrete improved the bond between the GFRP and concrete interface compared to that of normal concrete.

**Figure 11:**
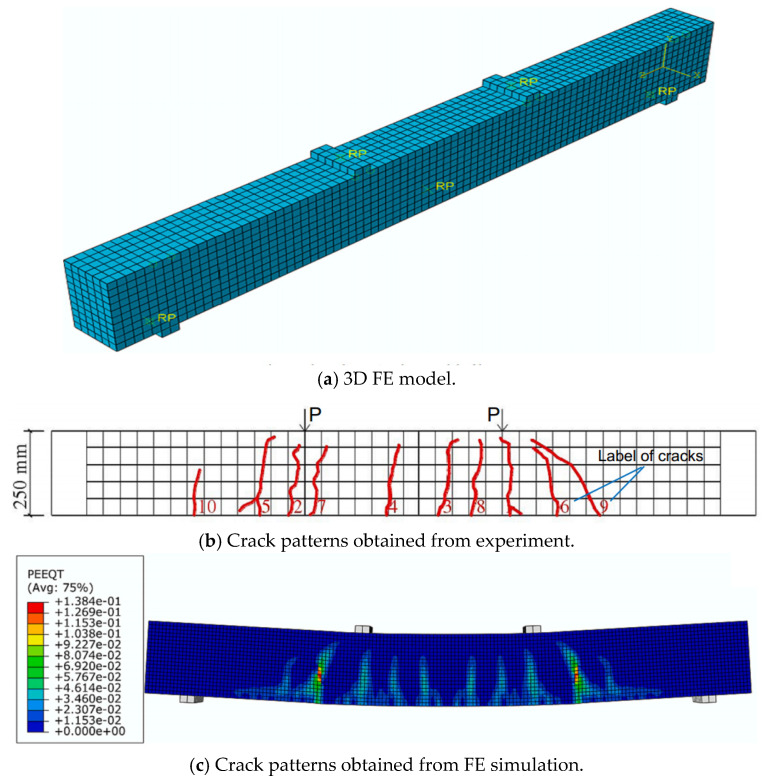
Three-dimensional modeling of FRP RC beams [19].

**Figure 12:**
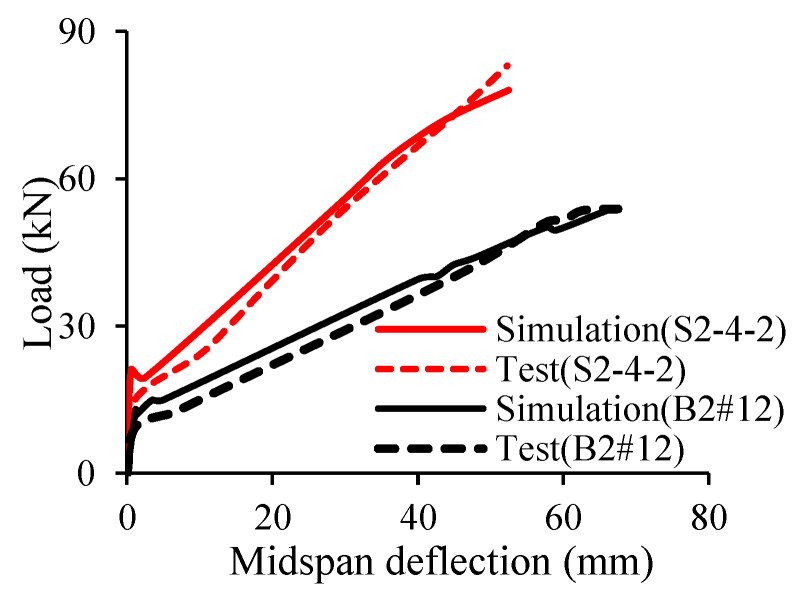
Comparison between test and simulation [19].

Chen et al. [19] constructed 3D numerical model in ABAQUS to analyze the effects of reinforcement ratio on the tension stiffening of FRP RC beams. In their study, concrete was simulated by 8-node solid elements, namely C3D8R, while reinforcing bars were modeled by truss elements, namely T3D2. The constitutive laws of materials were also rigorously considered in the study. For concrete, the stress–strain relationship given in the Chinese design standard GB50010-2010 [130] was applied. It was implemented into ABAQUS by using concrete damage plasticity model. In the case of FRP bars, their behavior was considered linear until its rupture. The results from numerical simulation including the crack patterns and load–deflection curves agreed well with those from the experiments as shown in Figure 11 and Figure 12. Their study introduced the correction factor and a method to determine the relationship between the correction factor and reinforcement ratio. After verifying the results obtained by this method with those from the experiments, it was suggested that their proposed method could be used for various reinforcement ratios.

Shen et al. [46] conducted a 2D numerical analysis in a commercial finite element software, namely DIANA v10.4, to investigate the behavior of BFRP-reinforced concrete beams and to explore the effects of the CY blocks on the beam’s failure mechanism. In their study, concrete was simulated by plane stress shell elements, namely CQ16M, while FRP bars were represented by truss elements that were embedded to the surrounding concrete. To reflect the actual response of materials under loading conditions, their stress–strain relationships were considered. For concrete, the smear crack model was applied. FRP bars were considered as elastic materials. The stress–strain relationship of the CY block was bilinear. The results of their numerical simulation agreed well with those obtained from experiments in terms of the ultimate load, the crack patterns, and the load–displacement relationship. Their study showed that the CY block was able to increase the load-carrying capacity of the FRP RC beams by 16%. In addition, the ductility was also improved up to 29%.

**Figure 13:**
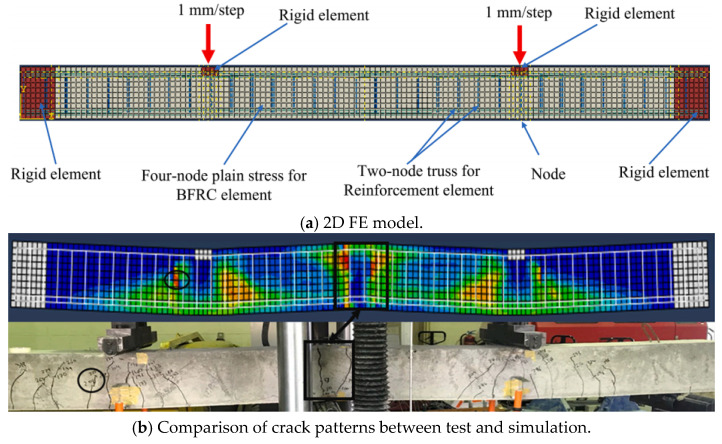
Continuous FRP RC beams [32].

**Figure 14:**
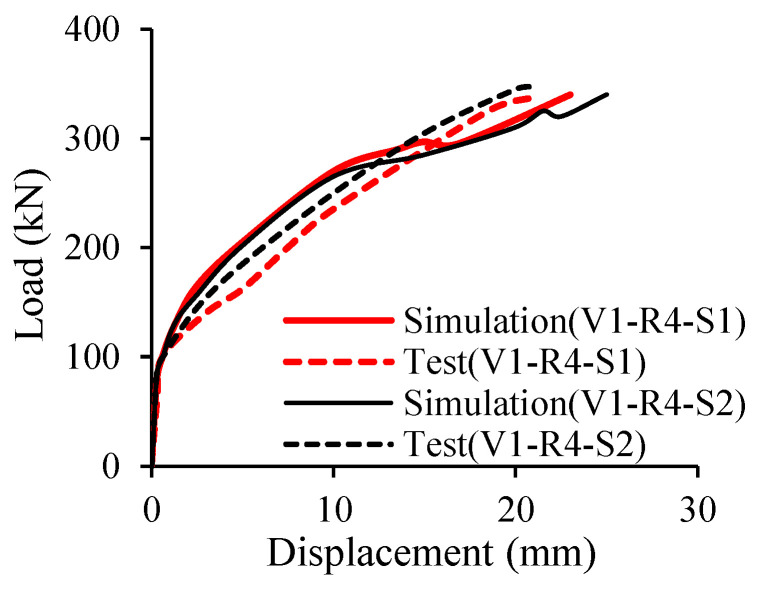
Comparison between test and simulation [32].

Abushanab et al. [32] applied ABAQUS to conduct 2D numerical simulation of BFRP-reinforced continuous concrete beams. In their study, concrete was modeled using 4-node solid elements, while reinforcing bars were simulated by truss elements. To capture the practical behavior of the tested beams, appropriate constitutive models for materials were employed. For concrete, the stress–strain curve was implemented into ABAQUS by using concrete damage plasticity model. In the case of FRP, linear stress–strain relationship was considered. The results from numerical simulations agreed quite well with those from experiments as shown in Figure 13 and Figure 14. The basalt macro-fibers (BMF) added to the concrete beams were able to increase the ductility of the beams and reduced the crack widths (up to 62%) under loading conditions. Their study also found that changing the reinforcement ratio could influence the values of sagging and hogging moments.

Zinkaah et al. [51] conducted 3D numerical analysis in ABAQUS to assess the performance of FRP-reinforced geo-polymer concrete beams under four-point bending. In their study, concrete was modeled by solid elements, namely C3D8R, while truss elements were used to simulate FRP reinforcing bars. In addition, the constitutive laws of concrete and FRP were also incorporated into the numerical model. The stress–strain relationship of concrete proposed by Hognestad et al. [106] was implemented into ABAQUS by utilizing concrete damage plasticity model. For FRP, the stress–strain relationship was considered linear. The results of their numerical model demonstrated good agreement with those obtained from experiments in terms of the ultimate load, the failure mode, and the crack patterns. Their study also indicated that the concrete compressive strength had a significant influence on the ultimate load of the beams without web reinforcement. In addition, they recommended that the design equations given by CSA S806 and ACI 440.1R should be revised as they underestimated the load-carrying capacity of FRP-reinforced geo-polymer concrete beams.

Metwally [43] investigated the effects of parameters such as the reinforcement ratio, the effective depth, and the compressive strength of concrete on the ultimate load, the failure mode and the crack propagation of GFRP RC deep beams by conducting 3D numerical analysis in ABAQUS. In his study, concrete was modeled by solid elements, namely C3D8R, while truss elements, namely T3D2, were applied to simulate GFRP bars. After the verification, the load–displacement curves and the crack patterns of the FE model showed good agreement with those obtained from experiments. The numerical results demonstrated that the strain distribution along the FRP bars was uniform when applied loads increased. In addition, the analysis also illustrated that the deflection of GFRP RC deep beams was two to four times higher than that of beams reinforced with CFRP.

Domenico et al. [124] developed a feasible calculation tool based on the FE method to predict the ultimate load and the failure mechanism of FRP RC beams and slab. The results of their study demonstrated that the computational tool is quite useful since it could capture the load-carrying capacity, the damaged zones, and the failure mode of FRP RC beams. However, it was unable to simulate the cracks, the interface problems, and the creep of the concrete.

Cai et al. [123] investigated the behavior of BFRP-reinforced engineered cementitious composite (ECC) and traditional concrete by conducting numerical simulation in ATENA. Their study discovered that BFRP ECC beams were superior to BFRP concrete beams with higher ductility, higher load-carrying capacity, and better crack controlling ability.

Mohamed et al. [125] conducted 2D FE analysis to evaluate and modify the strut-and-tie model of GFRP RC deep beams. The results of the analysis agreed well with those of the experiments. In addition, the vertical reinforcement did not really affect the resistance of the beams, but they found that the size of the loading plate had a strong influence on the load-carrying capacity of the beams. The modified strut-and-tie model demonstrated good agreement with the current design codes and provisions.

Yang et al. [126] conducted an FE analysis to evaluate the damage distribution, the energy dissipation, and the strain distribution of GFRP RC beams. The failure mode and the load-carrying capacity of the beams predicted by their simulation showed good agreement with those obtained from experiment. However, the bond between the GFRP bars and the surrounding concrete was ignored. This matter should be considered in future studies.

### 4.2. Discrete Element (DE) Analysis

**Figure 15:**
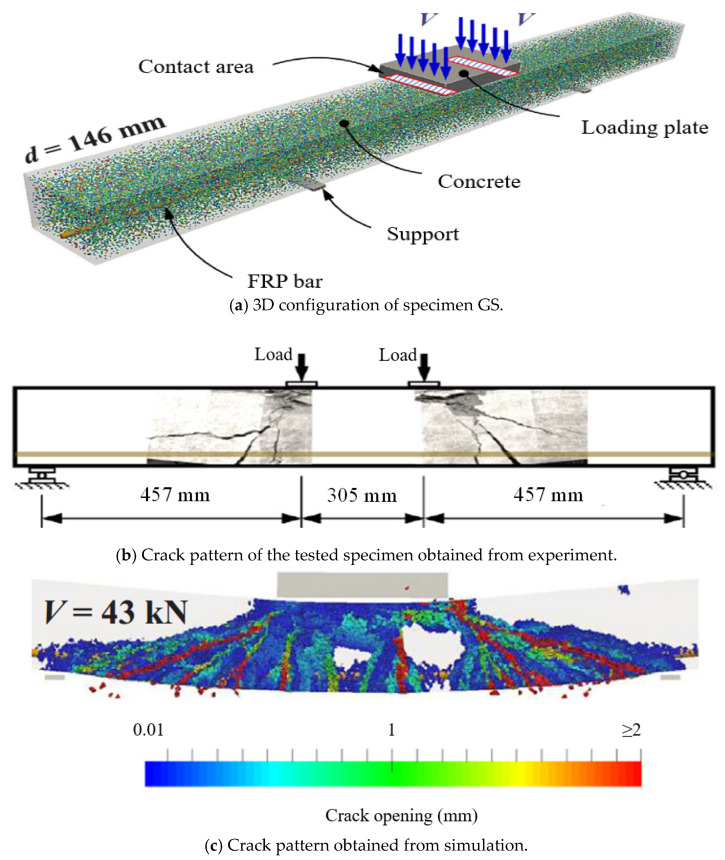
Modeling of FRP RC beams by DEM [127].

**Figure 16:**
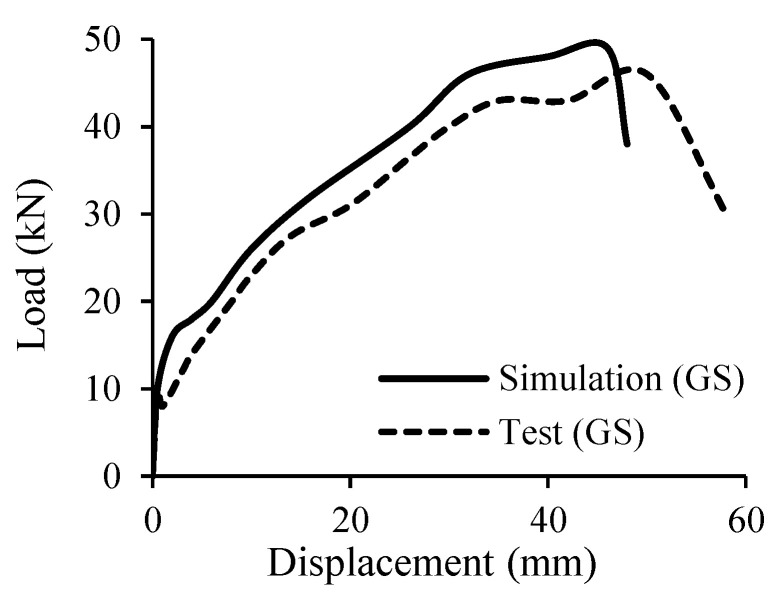
Comparison between test and simulation [127].

Besides FE analysis, DE analysis has also been utilized widely to study the fracture of concrete [131,132,133,134]. However, the application of DE analysis to analyze the behavior of FRP RC beams is still very limited. According to the current information, the authors can find only one study conducted by Khodaie et al. [127] about this research topic. In their study, the Lattice Discrete Particle Element (LDPM) was applied to build the numerical model in which aggregates in the concrete were modeled by spherical elements while the surrounding mortar matrix was analyzed by the polyhedral cells. The constitutive law of concrete is also assigned to these elements. Regarding the GFRP bars, the linear stress–strain relationship was considered. The results of their simulation agreed well with those from the experiments in terms of the stiffness, the load-carrying capacity, and the crack patterns (Figure 15 and Figure 16), which demonstrated that their developed numerical model was reliable.

### 4.3. Artificial Intelligence/Machine Learning (AI/ML)

In addition to FE analysis and DE analysis, AI/ML is a novel and efficient approach to predict the behavior of FRP RC beams by taking advantage of the database collected from previous studies. Several ML approaches such as random forest (RF), extreme gradient boosting (XGBoost), and artificial neural network (ANN) have been applied in some studies to compute the ultimate load-carrying capacity of FRP RC beams [39,128,129,135,136].

**Figure 17:**
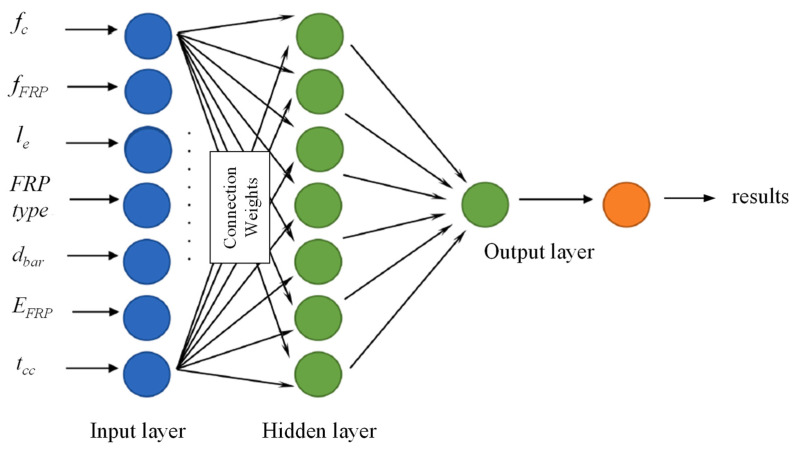
ANN for predicting the bond strength between FRP and concrete [128].

Concha [128] employed ANN to determine the bond strength between concrete and FRP bars based on initial inputs such as the concrete compressive strength, the FRP tensile strength, the section dimensions, and the elastic modulus as depicted in Figure 17. According to the results of the study, it has been found that concrete compressive strength had the strongest influence on the bond strength between the FRP and concrete interface. Other parameters including the FRP embedment length, the elastic modulus, and the thickness of the concrete cover layer were less influential.

Murad et al. [129] utilized gene expression programming to anticipate the flexural capacity of FRP RC beams based on the database of 116 tested specimens. Various parameters including the concrete compressive strength, the section dimensions, the FRP tensile strength, and the elastic modulus were used. The results of their study demonstrated that the simplest GEP model can predict the moment capacity of FRP RC beams very well as the value of *R*-square was relatively high (over 0.95).

Guo et al. [39] applied ML to assess and optimize the ultimate moment of compression yielded FRP RC beams with T-section. In their study, the load-carrying capacity, the ductility, and the optimal design of the investigated beams were obtained by a model in which ANN, the support vector machine, and the Gaussian process regression are integrated. The results of their study demonstrated that ML could be used to analyze the behavior and optimize the design of compression yielded FRP RC beams with T-section.

## 5. Design Guidelines

**Figure 18:**
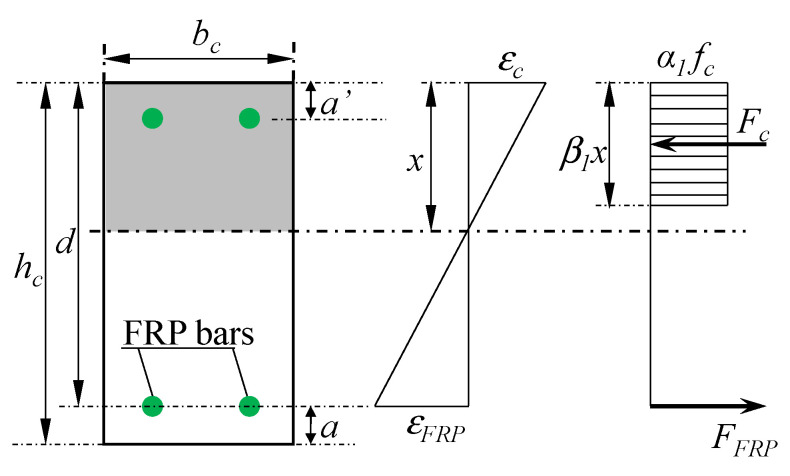
Distribution of concrete strain (ε) and stress (*α*_1_*f_c_*) of rectangular sections.

The design of FRP RC beams has been given various national design standards. In this section, three design documents, ACI 440.1R [137], CEB-FIB [138], and CSA S806 [139], are summarized as shown in Table 8. According to these design documents, the section of concrete beams is assumed to be plane under loading conditions, the concrete strain is linearly distributed along the section and the concrete compressive stress is converted to the equivalent rectangular stress block (see Figure 18) using coefficients such as *α*_1_, *β*_1_, *λ*, and *η* given in Table 8. The main difference between these design documents is that ACI 440.1R and CEB-FIB allow both concrete crushing and FRP rupture to occur, but FRP rupture is not permitted in CSA S806. In addition, CSA S806 and CEB-FIB use the material safety factors when calculating the design moment, while ACI 440.1R applies the reduction factor to obtain the design moment. Three design documents determine the type of failure mode based on balanced reinforcement ratio (*ρ_fb_*) and the reinforcement ratio *ρ_f_ = A_f_/(b_c_d)*, where *A_f_* is the area of FRP reinforcement (mm^2^), *b_c_* is the width of the section (mm), and *d* is the effective depth of the section (mm). If *ρ_f_ >ρ_fb_* then the failure mode is concrete crushing. Otherwise, the failure mode is FRP rupture. Based on the force balanced equation, the load-carrying capacity of the FRP RC beams can be determined.

**Figure 19:**
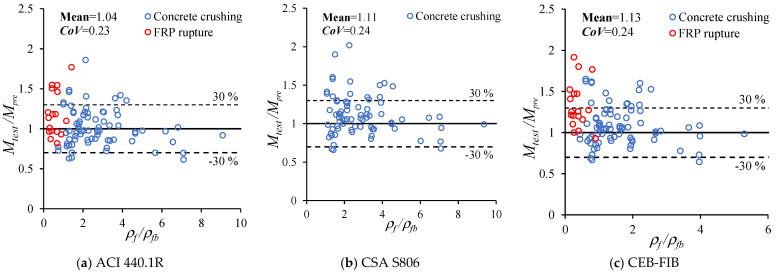
*M_test_/M_pre_* ratios of presented design standards.

To evaluate the performance of the mentioned design standards, 93 FRP RC beams [140] have been collected from the previous experimental studies, and they are presented in Table 9 in which *b_c_* is the width of the beam (mm), *h_c_* is the height of the beam (mm), *f_c_* is the compressive strength of concrete (MPa), *A_f_* is the total area of FRP bars (mm^2^), *E_f_* is the elastic modulus of FRP bars (GPa), *f_f_* is the tensile strength of FRP bars (MPa), and *M_test_* is the ultimate moment obtained from experiments (kNm). Figure 19 illustrates the comparison between results obtained from the presented design standards and experiments. As can be seen from this figure, it is obvious that the values of *M_test_/M_pre_* fluctuate in a wide range from 0.7 to 2.0. However, most of them vary from 0.7 to 1.3. Among the three design standards, ACI 440.1R gives the best prediction of ultimate moment of FRP beams since the mean and *CoV* are 1.04 and 0.23, respectively.

## 6. Conclusions and Recommendations

This paper provides an overview of studies on the flexural behavior of FRP RC beams. In the paper, current experimental and numerical studies on beams cast from various kinds of concrete and different types of FRP bars have been collected and summarized. The key parameters investigated and main findings of these studies are also given in the paper. In addition, available design guidelines are presented and evaluated. Based on the results of the studies, some conclusions and recommendations are deduced as follows:

(1) Many experimental and numerical studies on FRP RC beams with the consideration of different types of concrete and FRP have been carried out. Based on these studies, 93 tested beams have been collected, and it can be a good source of reference for other future studies.
(2) The application of the AI/ML and the DE method to investigate the behavior of FRP RC beams are still limited. More studies using these approaches can be conducted.
(3) Although numerous experimental studies and studies using FE simulation have been found, few studies consider the effect of the bond and the radial stress between concrete and FRP bars on the behavior of FRP RC beams. In addition, research focusing on the behavior of FRP RC deep beams and FRP RC continuous beams is still limited.
(4) Almost all studies focus on beams with rectangular sections; beams with other shapes of section can be investigated. In addition, most studies investigate the strength limit state of FRP RC beams; few research explores the serviceability performance of FRP RC beams.
(5) Three prevalent design standards of FRP RC beams are summarized in the study. Among these standards, ACI 440.1R can give the best prediction of moment capacity of FRP RC beams as the *M_test_/M_pre_* ratio has the mean and *CoV* of 1.04 and 0.23, respectively.

## Figures and Tables

**Table 1:** Types of fiber-reinforced polymers (FRP) and their basic mechanical properties.

Types of Bars	Density (kg/m^3^)	Tensile Strength (MPa)	Young Modulus (GPa)	Price (USD/kg)	Melting Point (°C)	Number of Specimens
GFRP	1250–2500	483–4890	35–86.9	1.75	880	67 (72%)
CFRP	1500–2100	600–3900	37–784	11.7	1600	22 (23.7%)
BFRP	2630–2800	upto 4840	40–110	7.6	1500–1700	2 (2.15%)
AFRP	1250–1450	1700–3600	41–175	-	500	2 (2.15%)
Steel	7850	250–690	200	0.6	1300–1600	-

**Table 2:** Physical properties of glass fibers.

Fiber	Density (g/cm^3^)	Tensile Strength (MPa)	Young’s Modulus (GPa)	Elongation (%)	Coefficient of Thermal Expansion (10^−7^/°C)
A-glass	2.44	3310	68.9	4.8	73
AR-glass	2.7	3241	73.1	4.4	65
C-glass	2.52	3310	68.9	4.8	63
D-glass	2.11–2.14	2415	51.7	4.6	25
E-glass	2.58	3445	72.3	4.8	54
EGR-glass	2.72	3445	80.3	4.8	59
R-glass	2.54	4135	85.5	4.8	33
S_2_-glass	2.46	4890	86.9	5.7	16

**Table 4:** Stress–strain relationships of concrete.

σ–ε Relationship	Methodology	Source
$\sigma =f_{c}\frac{Ax+\left(D-1\right)x^{2}}{1+\left(A-2\right)x+Dx^{2}}$ $A=E_{c}\varepsilon_{o}/f_{c},\;\;x=\varepsilon /\varepsilon_{o}$ $E_{c}=5975\sqrt{f_{c}}$ $D=0.65-7.25f_{c}\times 10^{-3}$	Empirical approach	[109]
$f=f_{c}\frac{\varepsilon }{\varepsilon_{o}}\frac{n}{n-1+{\left(\varepsilon /\varepsilon_{o}\right)}^{n}}$ $n=0.4\times 10^{-3}f_{c}+1.0$	Numerical approach	[108]
$\sigma =\left\{\begin{array}{l} E_{c}\varepsilon -\frac{E_{c}\varepsilon^{2}}{2\varepsilon_{o}}\;\;\;\;\;\mathrm{if}\;\varepsilon \leq \varepsilon_{o}\\ f_{c}-\frac{0.8f_{c}\left(\varepsilon -\varepsilon_{o}\right)}{\varepsilon_{u}-\varepsilon_{o}}\;\mathrm{if}\;\varepsilon_{o}<\varepsilon \leq \varepsilon_{u}\\ 0.2f_{c}\;\;\;\;\;\;\mathrm{if}\;\varepsilon >\varepsilon_{u} \end{array}\right.$ $E_{c}=4700\sqrt{f_{c}},\;\;\varepsilon_{o}=2f_{c}/E_{c},\;\;\varepsilon_{u}=4\varepsilon_{o}$	Empirical approach	[28,107]
$\sigma_{c}=\left\{\begin{array}{l} f_{c}\left[2\left(\frac{\varepsilon }{\varepsilon_{o}}\right)-{\left(\frac{\varepsilon }{\varepsilon_{o}}\right)}^{2}\right]\;\mathrm{if}\;\varepsilon \leq \varepsilon_{o}\\ f_{c}-\frac{0.15f_{c}\left(\varepsilon -\varepsilon_{o}\right)}{\varepsilon_{u}-\varepsilon_{o}}\;\;\;\mathrm{if}\;\varepsilon_{o}<\varepsilon \leq \varepsilon_{u} \end{array}\right.$	Empirical approach	[106]
$\sigma =f_{c}\left[\frac{\beta \left(\varepsilon /\varepsilon_{o}\right)}{\beta -1+{\left(\varepsilon /\varepsilon_{o}\right)}^{\beta }}\right]$ $\beta =1/\left[1-f_{c}/\left(E_{c}\varepsilon_{o}\right)\right]$ $E_{c}=10200{\left(f_{c}\right)}^{1/3},\;\;\varepsilon_{o}=0.00078{\left(f_{c}\right)}^{1/4}$	Empirical approach	[110]

**Table 5:** The values of *α* and *β* [111].

Type of Surface	*α*	*β*
Sand coated with deformed surface	5.24	0.33
Deformed surface	2.57	0.54
Sand coated	3.33	0.41
Smooth surface	0.18	0.96

**Table 6:** Objectives and key parameters investigated in some previous studies.

Source	Key Investigated Parameters	Objectives of the Study	Type of Test
[48,51,92]	Type and strength of reinforcement, reinforcement ratio, stirrup ratio, concrete compressive strength.	Evaluate the flexural performance of beams reinforced with FRP in terms of crack patterns, deformability, and failure mode.	Four-point bending, three-point bending.
[25,94,104]	Type and strength of reinforcement, reinforcement ratio, surface of reinforcement, compressive strength of concrete.	Explore the bond between FRP bars and concrete. Investigate the behavior and deflection response of FRP-reinforced concrete beams.	Four-point bending, pull-out test.
[49,117]	Dosage of aggregates, reinforcement ratio, concrete compressive strength.	Investigate the flexural response of beams reinforced with FRP bars. Propose a model to predict the ultimate moment of FRP beams.	Four-point bending, compression test.
[37]	Type and strength of reinforcement, reinforcement ratio, cementitious composite thickness.	Assess the behavior of FRP-reinforced cementitious composite concrete beams in terms of crack width, ultimate load, and failure mode.	Four-point bending.
[26,30,34,40,44]	Type and strength of reinforcement, reinforcement ratio, type of concrete.	Investigate the flexural behavior and serviceability performance of BFRP-reinforced normal/high strength concrete beams.	Four-point bending.
[88,103]	Type and ratio of reinforcement, span length of beams, steel–fibers ratio, concrete compressive strength.	Investigate the cracking behavior of FRP-reinforced beams. Propose equations to predict crack widths of FRP RC beams.	Four-point bending.
[47,95,118]	Reinforcement ratio, concrete compressive strength.	Assess the behavior of FRP-reinforced beams in terms of crack width, ultimate load, and failure mode. Evaluate the design standards.	Four-point bending, three-point bending.
[36,93]	Type and strength of reinforcement, reinforcement ratio, surface of reinforcement, compressive strength of concrete.	Investigate the flexural behavior and serviceability performance of FRP-reinforced beams in terms of crack width, ultimate load, and failure mode. Evaluate the design standards.	Four-point bending.

**Table 7:** Typical numerical studies on FRP RC beams.

Refs.	Numerical Analysis Method	Types of Software	Modeling of Concrete	Parameters	Key Findings
[19,42,43,46,51,123,124,125,126]	Finite element analysis	ABAQUS v6.13, DIANA v10.4, ATENA v5.4, self-developed program	Concrete damage plasticity model, concrete smear crack model, Fracture plastic model.	Strength of concrete, transverse reinforcement ratio, reinforcement ratio, type of FRP bars, compression yielded block, shear span-to-depth ratio, shear reinforcement, engineering cementitious concrete, and size of loading-plate.	Transverse reinforcements transfer stresses to flexural reinforcements, and they can affect the crack patterns. The bond quality of GFRP in high-strength concrete is higher than that in normal-strength concrete, CY block can increase the strength and ductility of the beam up to 30%, Compressive strength affects the capacity of beams without web reinforcement significantly, BFRP ECC beams were superior to BFRP concrete beams with higher ductility, higher load-carrying capacity, and better crack controlling ability.
[127]	Discrete element analysis	Self-developed program	Using the stress–strain relationship of concrete.	Reinforcement ratio, section dimension, and size of loading plate.	The developed program predicted the behavior of FRP RC beams well.
[39,128,129]	AI/ML	Self-developed program	Not used	Concrete compressive strength, section dimensions, FRP tensile strength, and elastic modulus.	The developed AI/ML model predicted the capacity of FRP RC beams well.

**Table 8:** Summary of prevalent design standards for FRP-reinforced concrete beams.

	ACI 440.1R	CSA S806	CEB-FIB
*ρ_fb_*	$\rho_{fb}=0.85\beta_{1}\frac{f_{c}}{f_{fu}}\frac{E_{f}\varepsilon_{cu}}{E_{f}\varepsilon_{cu}+f_{fu}}$ If *f_c_* ≤ 28 MPa: β=0.85 If *f_ck_* > 28 MPa: $\beta =\max\left[\left(0.85-0.05\times \frac{f_{c}-28}{7}\right),0.65\right]$ $\varepsilon_{cu}=0.003$	$\rho_{fb}=\alpha_{1}\beta_{1}\frac{\phi_{c}}{\phi_{f}}\frac{f_{c}}{f_{fu}}\frac{E_{f}\varepsilon_{cu}}{E_{f}\varepsilon_{cu}+f_{fu}}$ $\alpha_{1}=0.85-0.0015f_{c}\geq 0.67$ $\beta_{1}=0.97-0.0025f_{c}\geq 0.67$ $\phi_{c}=0.6,\;\;\phi_{f}=0.75$ $\varepsilon_{cu}=0.0035$	$\rho_{fb}=\frac{0.81\left(f_{c}+8\right)\varepsilon_{cu}}{f_{fu}\left(\frac{f_{fu}}{E_{f}}+\varepsilon_{cu}\right)}$ If *f_c_* < 50 MPa: $\varepsilon_{cu}=0.0035$ If *f_c_* ≥ 50 MPa: $\varepsilon_{cu}=2.6+35{\left[\left(90-f_{c}\right)/100\right]}^{4}$
*M_CR_* _(Concrete_ _crushing)_	$M_{CR}=\phi A_{f}f_{f}\left(d-\frac{a}{2}\right)$ $a=\frac{A_{f}f_{f}}{0.85f_{c}b}$ $f_{f}=E_{f}\varepsilon_{cu}\frac{\beta_{1}d-a}{a}$ $\phi =\left\{\begin{array}{c} 0.55\;\;if\;\rho_{f}\leq \rho_{fb}\\ 0.3+0.25\frac{\rho_{f}}{\rho_{fb}}\;if\;\rho_{fb}<\rho_{f}<1.4\rho_{fb}\\ 0.65\;\;if\;\rho_{f}\geq 1.4\rho_{fb} \end{array}\right.$	$M_{CR}=\phi_{f}A_{f}f_{f}\left[d-\frac{a}{2}\right]$ $f_{f}=\left\{\sqrt{\frac{{\left(E_{f}\varepsilon_{cu}\right)}^{2}}{4}+\frac{\phi_{c}\alpha_{1}\beta_{1}f_{c}}{\phi_{f}\rho_{f}}E_{f}\varepsilon_{cu}}-\frac{E_{f}\varepsilon_{cu}}{2}\right\}$ $a=\frac{\phi_{f}}{\phi_{c}}\frac{A_{f}f_{f}}{\alpha_{1}f_{c}b}$	$M_{CR}=\eta f_{cd}bd^{2}\left(\lambda \xi \right)\left(1-\frac{\lambda \xi }{2}\right)$ $f_{cd}=\frac{f_{c}}{\gamma_{c}}$ $\lambda =\left\{\begin{array}{c} 0.8\;\;\;\;if\;f_{c}\leq 50\;\mathrm{Mpa}\\ 0.8-\left(\frac{f_{c}-50}{200}\right)\;if\;50<f_{c}\leq 90\;\mathrm{Mpa} \end{array}\right.$ $\eta =\left\{\begin{array}{c} 1.0\;\;\;\;if\;f_{c}\leq 50\;\mathrm{Mpa}\\ 1.0-\left(\frac{f_{c}-50}{200}\right)\;if\;50<f_{c}\leq 90\;\mathrm{Mpa} \end{array}\right.$ $\xi =\frac{x}{d}=\frac{\varepsilon_{cu}}{\varepsilon_{f}+\varepsilon_{cu}}$ $\varepsilon_{f}=\frac{-\varepsilon_{cu}+\sqrt{{\varepsilon_{cu}}^{2}+\frac{4\eta f_{c}\lambda \varepsilon_{cu}}{\gamma_{c}\rho_{f}E_{f}}}}{2}$
*M_FR_* _(FRP rupture)_	$M_{FR}=\phi A_{f}f_{fu}\left(d-\frac{\beta_{1}c_{b}}{2}\right)$ $c_{b}=\left(\frac{\varepsilon_{cu}}{\varepsilon_{cu}+\varepsilon_{fu}}\right)d$	Not allowed	$M_{FR}=\frac{A_{f}f_{fu}d}{\gamma_{f}}\left(1-\frac{\xi }{2}\right)$ $\gamma_{f}=1.3$ $\xi =\frac{x}{d}=\frac{\varepsilon_{c}}{\varepsilon_{fu}+\varepsilon_{c}}$

**Table 9:** Summary of collected beams.

Source	*b_c_* (mm)	*h_c_* (mm)	*f_c_* (MPa)	*A_f_* (mm2)	*E_f_* (GPa)	*f_f_* (MPa)	*M_test_* (kNm)
Benmokrane et al. [18]	200	300–550	43–55	573	42–49	641–689	50.6–181.5
Thériault et al. [47]	130	180	46.2–97.4	237.7–475.3	38	773	19.7–29.5
Thiagarajan [94]	152.4	152.4	43.9–53.3	63.3–142.4	140	1900	10.2–17.5
Toutanji et al. [95]	180	300	35	253.4–506.7	40	695	59–71
Ashour [17]	150	200–300	34–59	56.6–113.1	38	650	5.9–16.8
He et al. [119]	150	300	21.3–33.1	226.9–314	52	1230	46.6–66.8
Lau et al. [120]	280	380	33.9–42.5	339.3–1963.5	38–40.2	582–603	80.4–237.9
Kassem et al. [93]	200	300	39.1–40.8	254.5–1013.4	36–122	617–1988	70.9–90.4
Escorcio et al. [118]	250	400	47.9–48.2	339–2455	60	1100–1350	85.9–206.2
El-Nemr et al. [36]	200	400	29–48	258–1194	46.4–69.3	666–1639	81–171
Ahmed et al. [33]	110	300	20–50	28.27–169.7	148	2000	20.9–67.9
Maranan et al. [99,121]	200	300	38.2	520–992.8	62.6 – 65.6	1105–1312	91.4–104.8

## Data Availability

No new data were created or analyzed in this study. Data sharing is not applicable to this article.
